# Temporal changes in the bacterial community of animal feces and their correlation with stable fly oviposition, larval development, and adult fitness

**DOI:** 10.3389/fmicb.2014.00590

**Published:** 2014-11-10

**Authors:** Thais A. Albuquerque, Ludek Zurek

**Affiliations:** ^1^Department of Entomology, College of Agriculture, Kansas State UniversityManhattan, KS, USA; ^2^Department of Diagnostic Medicine and Pathobiology, College of Veterinary Medicine, Kansas State UniversityManhattan, KS, USA

**Keywords:** stable fly, oviposition, development, fitness, horse feces, bacteria, diversity, richness

## Abstract

Stable flies are blood-feeding insects with a great negative impact on animals world wide. Larvae develop primarily in animal manure and bacteria are essential for larval development; however, the principle of this dependence is not understood. We hypothesized that as the microbial community of animal manure changes over time, it plays an important role in stable fly fitness. Two-choice bioassays were conducted using 2 week old horse manure (control) and aging horse manure (fresh to 5 week old) to evaluate the effect of manure age on stable fly oviposition. Our data showed that fresh feces did not stimulate oviposition and that the attractiveness increased as manure aged but started to decline after 3 weeks. Bioassays assessing the effect of manure age at the time of oviposition on larval development demonstrated that 1–3 week old manure supported larval development significantly better than fresh, 4, and 5 week old manure. In addition, adult fitness (body size) was significantly higher in flies from 1 and 2 week old manure comparing to that of all other treatments. Analysis of the bacterial community of aging horse manure by 454-pyrosequencing of 16S rDNA revealed a great reduction in bacterial diversity and richness from fresh to 1–5 week old manure and a major shift from strict anaerobes in fresh manure to facultative anaerobes and strict aerobes in aged manure. Overall, the microbial community of 2 and 3 week old horse manure with its dominant bacterial taxa *Rhizobium, Devosia,* and *Brevundimonas* stimulated stable fly oviposition the most and provided a suitable habitat for larval development. These bacteria represent the candidates for studies focused on better understanding of stable fly – microbial interactions.

## INTRODUCTION

Stable flies (*Stomoxys calcitrans*) are important cosmopolitan blood-feeding pests of confined and pastured livestock. The annoyance caused by their painful bites keeps animals from feeding and consequently reduces their weight gain and/or milk production (Moon, 2002; Taylor et al., 2012). The annual economic losses caused by stable flies in cattle industry in the United States were estimated at $2.2 billion (Taylor et al., 2012).

The use of insecticides to control stable flies, especially on pastures, is not effective, and the most important approach is sanitation and manure management to minimize larval developmental sites (Broce, 2006). Stable fly oviposition and larval development sites include various types of organic decomposing substrates but animal manure is most abundant and important (Meyer and Petersen, 1983; Axtell, 1986; Skoda et al., 1991). The stable fly development from eggs, through three larval stages, pupa and adult typically takes 3–4 weeks (Broce and Haas, 1999). The age of cattle manure was shown to influence stable fly oviposition preference and high numbers of gravid females visiting manure that aged for at least 2 weeks were reported (Broce and Haas, 1999). Female oviposition did not correlate with manure pH, osmolality, CO_2_, temperature, or amonia production (Broce and Haas, 1999); however, adult emergence correlated with moisture content, pH, ammonia concentration, electrical conductivity, total carbon concentration, and microbial respiration rate (Wienhold and Taylor, 2012). Due to bacterial decomposition, characteristics of a substrate, including litter and manure change over time considerably (Couteaux et al., 1995; Broce and Haas, 1999). Furthermore, it has been demonstrated that an active bacterial community is essential for stable fly larval development (Lysyk et al., 1999, 2002; Rochon et al., 2004; Romero et al., 2006) and also likely produces cues for stable fly oviposition (Romero et al., 2006; Gilles et al., 2008). Talley et al. (2009) reported a correlation between the concentration of fecal coliform bacteria around cattle hay feeding sites and high numbers of emerging stable flies. Interestingly, although cattle manure is much more abundant and available for stable fly development, horse manure was shown to be more attractive for stable fly oviposition than cattle manure (Jeanbourquin and Guerin, 2007). Furthermore, stable flies laid more eggs on horse manure with a complex bacterial community than on sterile manure and individual bacterial taxa such as *Serratia fanticola* and *Citrobacter freundii* isolated from horse manure stimulated stable fly oviposition and supported larval development significantly more than the sterile substrate (Romero et al., 2006). In general, insects have to reach a critical body weight to initiate the metamorphosis. The quality of the larval diet consumed during the 24 h period after the critical weight is achieved and before the molting hormone ecdysone is released is what determines the insect adult body weight (Davidowitz and Nijhout, 2004; Mirth and Riddiford, 2007). We hypothesized that as the microbial community grows and changes overtime, this will result in different oviposition responses and also reduce the availability of nutrients and consequently impact stable fly development and fitness.

Overall, the equine hindgut microbiota has received less attention comparing to that of ruminants and culture-independent methods characterizing the equine hindgut bacterial community are scarce and usually focus an animal health (Daly et al., 2001; Daly and Shirazi-Beechey, 2003; Yamano et al., 2008; Shepherd et al., 2012). It was shown that the resident microbial community of the horse hindgut comprises the phyla *Firmicutes*, *Verrucomicrobia*, *Proteobacteria*, *Bacteroidetes*, and *Spirochaetes* (Daly et al., 2001; Shepherd et al., 2012).

Studies on how the horse fecal bacterial community changes overtime and how that may impact behavior and fitness of insects that develop in horse manure are lacking. The purpose of this study was to assess the changes in the bacterial community of aging horse manure and how that may be reflected in stable fly oviposition, larval development, and adult fitness.

## MATERIALS AND METHODS

### MANURE COLLECTION

Manure was collected from the Kansas State University Horse Facility, from a pen with 16 two year old horses on a diet consisting of 25% of grain (Purina Strategy-Professional formula GX^®^, Purina, Gray Summit, MO) and 75% of alfalfa hay. Horses did not receive any treatments (e.g., insecticides, dewormers, antibiotics) for at least 4 weeks before fecal collections.

Fresh (<10 min old) feces from the pen floor were collected in three 16 L plastic buckets (24cm × 24cm × 36 cm). Buckets were cleaned with hot water and soap (Palmolive original detergent®;, Colgate-Palmolive Company, New York, NY, USA), then sprayed with 70% ethanol and dried before use. Only the top 2/3 of each manure pad was collected to avoid soil contamination. Manure was taken to the laboratory, mixed by stirring, and divided equally into five 16 liters plastic buckets (24cm × 24cm × 36 cm) and closed with the plastic lid. Each lid had two round openings (5 cm diameter) covered with the filter paper to accommodate air exchange. Buckets were placed in an environmental chamber (26∘C, 40% RH, 15:9 L/D) to age for up to 5 weeks. Each week, one bucket was removed from the chamber and used for the experiment. In addition, one extra bucket of fresh manure was collected every week, for five consecutive weeks, aged for 2 weeks, and used as control.

### FLIES

Stable flies were from the laboratory colony maintained on sucrose water and citrated cattle blood. Flies were synchronized to lay eggs by selecting only adults that emerged within a period of 24 h. After seven days of daily *ad libitum* blood meals, synchronized flies ready to lay eggs were used for oviposition bioassays.

### OVIPOSITION ASSAY

Before each assay, the control manure (2 weeks old) was removed from the incubator, and 50 g/dish were distributed into 23 Petri dishes (60 × 150 mm; Fisher Scientific, Pittsburgh, PA, USA; 20 for individual bioassays and three for the group bioassays). Fifty grams of fresh manure (less than 2 h old) or aged manure (1–5 weeks) were also placed into 23 Petri dishes. For all assays, manure moisture was determined by measuring the dry weight of 10 g of each manure sample and then adjusted to 80% with sterile deionized water. Two-choice assays (control v aged manure) with individual flies (*n* = 20) and group flies (three groups of 30 flies) were conducted in plastic insect rearing cages (30 × 30 × 30 cm; BugDorm®;, MegaView, Taichung, Taiwan) kept in 26∘C and 14L: 10D light regime. Individual flies were allowed to oviposit (by placement of the dishes with manure) for 6 h on day 1 and for additional 6 h (with two new oviposition dishes; two technical replicates for the substrates) on day 2 to maximize the probability of egg laying. The group flies were allowed to oviposit for 2 h on day 1 and for additional 2 h on day 2. During assays, flies did not receive food or water. After oviposition, eggs were separated from manure by floatation in salt saturated water and counted. Each bioassay was replicated twice in separate experiments (biological replicates).

The chi-square test was performed to analyze the data on the number of flies laying eggs in either aged manure, control manure, or both (representing % ovipositing flies). Student’s *t*-test (StatPlus:mac, AnalystSoft Inc., 2009) was performed to determine significant differences between the mean number of eggs laid on aging and control manure.

### LARVAL DEVELOPMENT ASSAY

Plastic containers (*n* = 3) with 500 g of aging manure (from fresh to 5 weeks old) and containers with 500 g of the stable fly rearing larval medium (control) were used for larval development assays. The stable fly larval artificial medium was a mixture of 130 g of cattle feed (Calf Manna multi species performance supplement®;, Manna Pro, Chesterfield, MO, USA), 170 g of vermiculite, 500 g of wheat bran (Wingold Bakers Bran®;, Bay State Milling Company, Wichita, KS, USA) and 1.8 L of water. Plastic containers were washed with hot water and soap, sprayed with 70% alcohol, and dried before use. The moisture of each horse manure substrate was assessed as described above and adjusted to 80% before each experiment.

Five hundred stable fly eggs from the laboratory colony were placed in each container on the top of the substrate. Each container was closed with paper towel and rubber band. Containers were placed in the environmental chamber (26∘C, 40% RH, 15:9 L/D) and monitored daily for larval development and adult emergence. Freshly emerged adult flies were transferred to a plastic container, cooled down, and weighed. The data on survival to adult stage and development time were analyzed by ANOVA (PROC-GLM, SAS Institute 9.2).

### CORRELATION BETWEEN STABLE FLY BODY WEIGHT AND WING SIZE

To compare the size of stable flies in these experiments to that of wild flies, the wing size of 150 wild stable flies collected at the K-State horse facility (*n* = 50), dairy farm (*n* = 50), and cattle feedlot (*n* = 50) was manually measured. Another 140 freshly emerged stable flies from the laboratory colony had their wing size (mm) and fresh adult body weight (mg) measured. The wing size was measured from the base of the wing basicosta to the farthest point (close to the fourth vein) using a Leica MZ APO microscope. Linear regression was used to assess the correlation between fresh body weight and wing size (PROC REG, SAS Institute 9.2).

### ASSESSMENT OF THE BACTERIAL DIVERSITY BY 454 PYROSEQUENCING

Samples (∼10 g) of aging horse manure (from fresh to 5 weeks old) used in the oviposition assays were collected weekly (on the day of the two-choice experiment) and frozen in -80∘C for later analysis. Total genomic DNA was extracted from the fecal samples (0.5 g) using FastDNA®; SPIN kit (MP Biomedicals) following manufacturer’s instructions. The bacterial tag-encoded FLX-Titanium 16S rDNA amplicon parallel pyrosequencing and post sequencing processing were carried out at the Medical Biofilm Research Institute (Lubbock, TX, USA) as described by Dowd et al. (2008) and Middelbos et al. (2010). Briefly, primers extended from 27 F numbered in relation to the *Escherichia coli* 16S ribosomal gene were used. A single step reaction with 30 cycles was used and 0.5 U of HotStar HiFidelity Polymerase (Qiagen Inc., Valencia, CA, USA) was added to each reaction. Labeling and pyrosequencing was conducted as described in Dowd et al. (2008). Raw data from bacterial tag-encoded FLX amplicon pyrosequencing (bTEFAP) were screened and trimmed based upon quality scores and binned into individual sample collections. Sequence collections were depleted of chimeras using B2C2 software (Gontcharova et al., 2010). The resulting files were then depleted of short reads (<350 bp), reads with ambiguous base calls, reads with homopolymers >6 bp, and reads with lower than Q25 quality scores. Data were analyzed and interpreted using Sequencher 4.8 (Gene Codes) for alignment and sequence editing, MOTHUR (Schloss et al., 2009) for diversity and richness, and Blast2GO (http://www.blast2go.com) for the NCBI GenBank search.

## RESULTS

### OVIPOSITION ASSAYS WITH INDIVIDUAL FLIES

**FIGURE 1:**
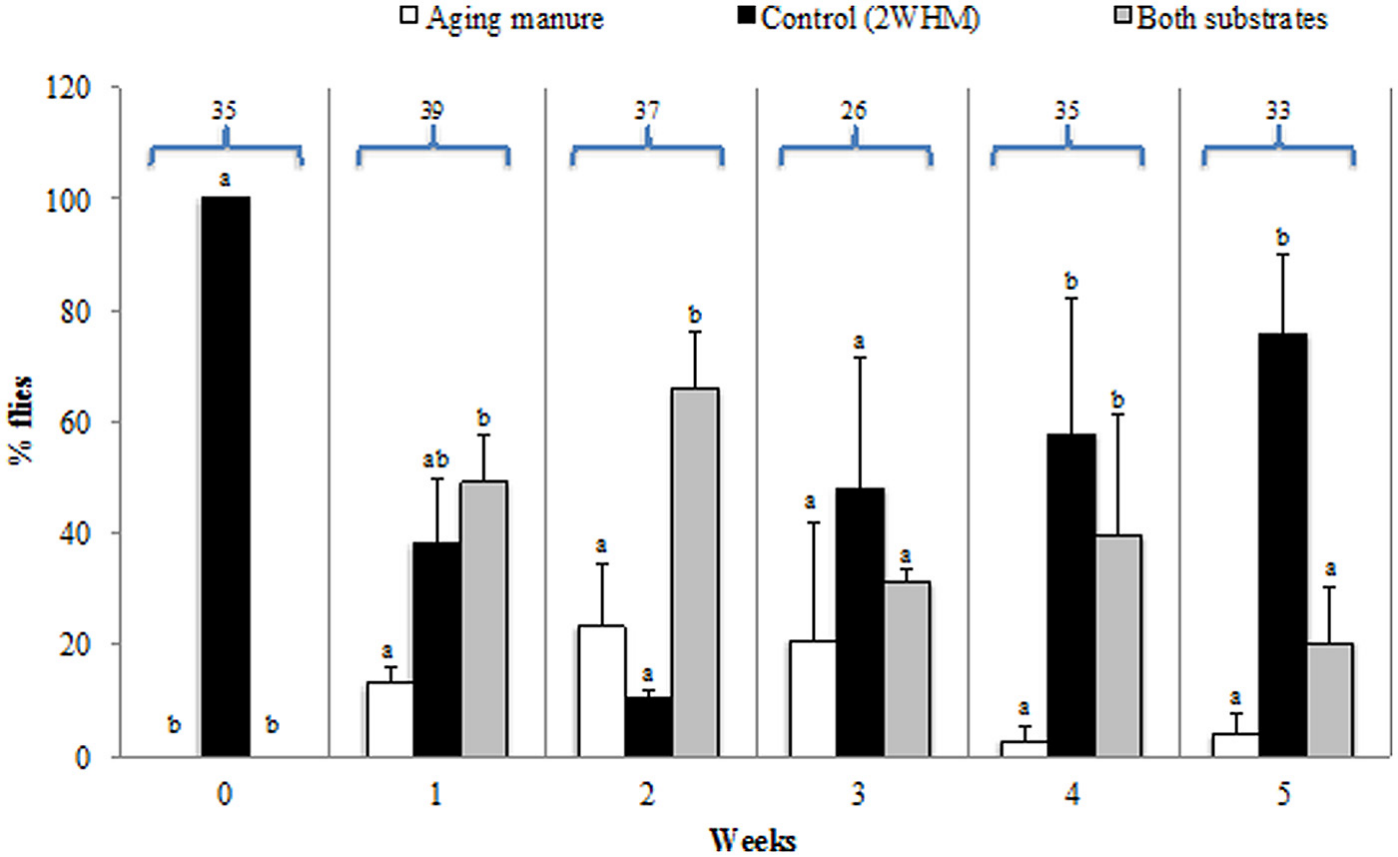
**Oviposition (% of flies) of individual stable flies on aging (fresh to 5 week old; white bars) or 2 week old horse manure (2 WHM; control; black bars).** Gray bars indicate % of flies ovipositing on both substrates (aging and control). Aging manure is fresh at week 0 and ages progressively to 5 weeks. Different letters indicate significant differences (*p* < 0.05) among the three different options within the same week. Numbers above bars are the total number of flies that oviposited in the two experimental replicates. Error bars are standard of mean.

Fresh manure did not stimulate stable fly oviposition and eggs were laid strictly on the control substrate (2 weeks old horse manure = 2 WHM; **Figure 1**; Table S1). When give a choice between aging (1–5 WHM) manure and control, flies started ovipositing on both types of manure but with a clear preference for 2 WHM (**Figure 1**; Table S1). In a choice between 1 WHM vs. control, 12.9% of the flies laid the eggs on the 1 WHM, 38.2% on control, and 48.9% on both substrates (*p* = 0.018). Some flies (20.8%) oviposited on 3 WHM, 48.2% on control, and 31.0% on both manure types (*p* = 0.15189). Very few flies (2.8%) oviposited on 4 WHM, while 57.8% preferred the control, and 39.4% chose to oviposit on both substrates (*p* = 0.00031). The oldest manure tested (5 WHM) did not stimulate oviposition and the majority (75.8%) of eggs were laid on the control manure (*p* = 0.00001; **Figure 1**; Table S1).

**FIGURE 2:**
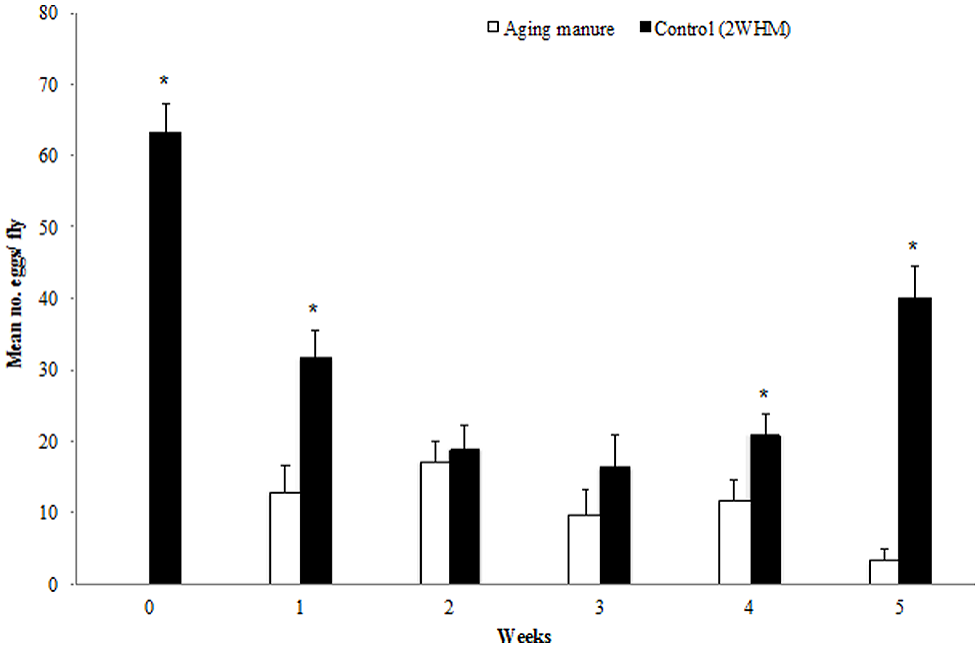
**Oviposition (number of eggs) of individual stable flies on aging and 2 week old horse manure (2 WHM; control).** Aging manure (white bar) is fresh at week 0 and ages progressively to 5 weeks. Stars indicate significant differences (*p* < 0.05) between the two choices within the same week. (*n* = 35, 39, 37, 26, 35, and 33, respectively. Error bars are standard of mean.

Overall, significantly more eggs per fly were laid on 2 WHM when the other choice was fresh manure (*p* = 0.00001), 1 WHM (*p* = 0.00104), 4 WHM (*p* = 0.02858), and 5 WHM (*p* = 0.00001). No significant difference was detected between the number of eggs per female that were laid on substrates 2 WHM vs. 2 HMM (*p* = 0.66794) and 2 WHM vs. 3 WHM (*p* = 0.24442; **Figure 2**).

### OVIPOSITION ASSAYS WITH GROUPS OF FLIES

Bioassays with 30 stable fly females per cage revealed the same trend as that with individual flies. Significantly more eggs per fly were laid on 2 WHM comparing to that of fresh (*p* = 0.00001) and 1 WHM (*p* = 0.02545; Figure S1). More eggs per female were also laid in 2 WHM when the other choice was 4 and 5 WHM although this difefrence was not significant (*p* = 0.15074; *p* = 0.27588, respectively; Figure S1). However, with this assay design, we could not determine how many females in a group oviposited.

### STABLE FLY SURVIVAL TO ADULT STAGE

**FIGURE 3:**
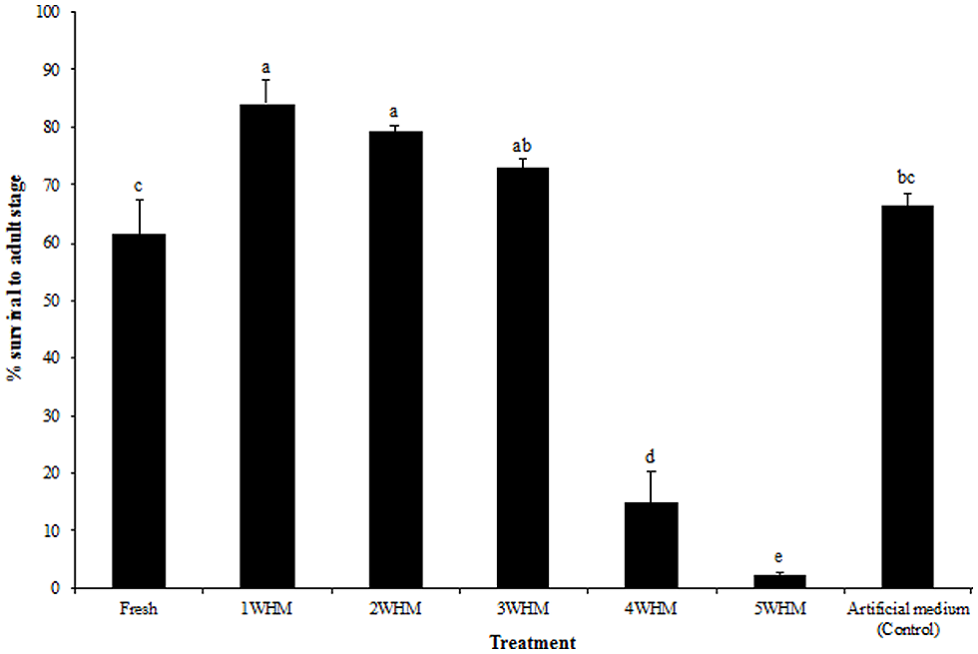
**Survival of stable flies from eggs to the adult stage in horse manure of different age [fresh to 5 weeks old (5WHM)] at oviposition and in the artificial medium.** Different letters indicate significant differences (*p* < 0.05) among treatments. (*n* = 1500). Error bars are standard of mean.

Stable fly survival to adult stage was not significantly different among 1, 2, and 3 WHM (*p* = 0.399; **Figure 3**). However, mortality was very high in 4 and 5 WHM, where only 14.9 and 2.1% of larvae respectively, developed into the adult stage. Interestingly, fresh manure supported larval development (61.3% survival) although into a significantly lesser extent (*p* = 0.0348) than that of 1–3 week old manure (**Figure 3**). Fly survival to adult stage in the control artificial medium was 66.4% and not significantly different (*p* = 0.2752) from that of fresh and 3 week old horse manure (**Figure 3**).

### LARVAL DEVELOPMENTAL TIME

Overall, as the manure age increased the development time of stable flies also increased (**Table 1**) and the correlation between the development time and manure age at the time of oviposition was strong (*r*^2^ = 0.82; Figure S4). Significant differences were detected among 1, 2, and 3 WHM (*p* < 0.0001); but not among 3, 4, and 5 WHM (**Table 1**). Interestingly, larval development in fresh manure was significantly longer comparing to that of 1 WHM (*p* < 0.0001). Larvae in the artificial medium developed the fastest (19.08 ± 0.04 days; **Table 1**). Stable fly daily emergence from each manure type is depicted Figure S2.

**Table 1:** Developmental time (mean ± standard of mean) of stable flies reared in horse manure of different age [fresh to 5 weeks old (5 WHM)] at the time of oviposition and the artificial medium.

Substrate	Developmental time (days)
Artificial Medium	19.08 ± 0.04^a^
Fresh	20.60 ± 0.08^c^
1 WHM	19.35 ± 0.07^b^
2 WHM	22.01 ± 0.07^d^
3 WHM	24.20 ± 0.07^e^
4 WHM	24.30 ± 0.15^e^
5 WHM	23.56 ± 0.41^e^

*Means followed by different letters are significantly different*.

### STABLE FLY BODY WEIGHT UPON EMERGENCE

**FIGURE 4:**
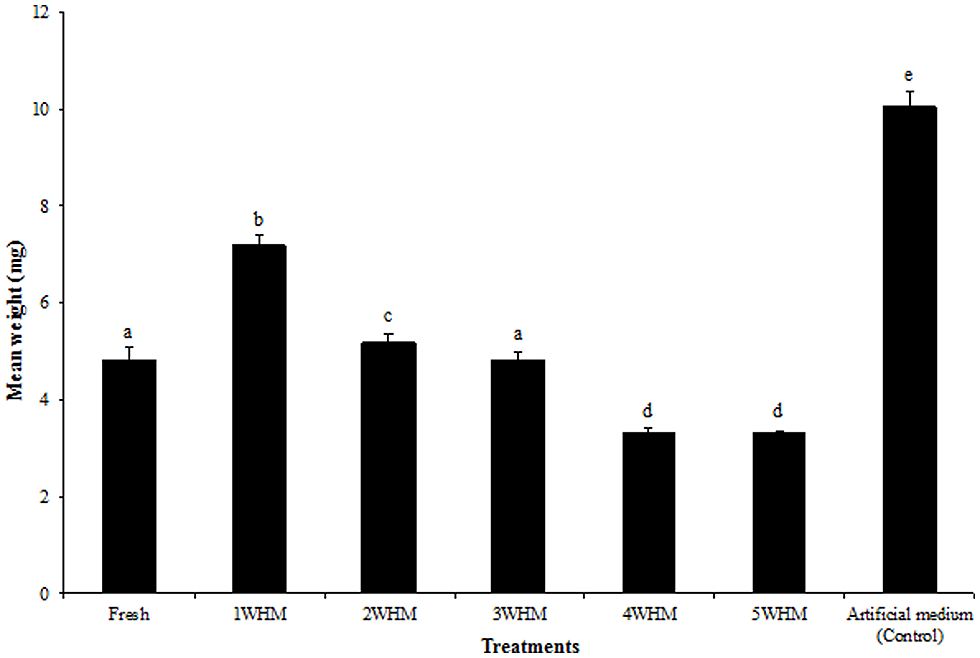
**Mean body weight of newly emerged adult stable flies that started development in horse manure of different age [fresh to 5 weeks old (5WHM)] and in the artificial medium.** Different letters indicate significant differences (*p* < 0.05) among treatments. (*n* = 919, 1262, 1190, 1097, 224, 32, and 2987, respectively). Error bars are standard of mean.

One weeks old horse manure at the time of oviposition generated significantly heavier (*p* = 0.0398) adult flies comparing to that of all other manure treatments (**Figure 4**). Larvae that started developing in fresh manure and 3 WHM resulted in flies weighing on average 4.80 and 4.87 mg, respectively, with no statistically significant difference (*p* = 0.1610). Overall, there was a strong negative correlation (*r*^2^ = -0.72) between the stable fly body weight and the age of the substrate (Figure S5). 4 and 5 WHM at the time of oviposition produced relatively light flies (3.38 and 3.36 mg respectively), with no statistically significant difference (*p* = 0.9167; **Figure 4**). The heaviest flies emerged from the artificial medium (control) with the overall mean body weight of 10.05 mg (**Figure 4**). The fresh adult body weight upon emergence from the same type of substrate was typically higher for flies that emerged earlier and declined for the adults emerging later (Figure S3).

### WING SIZE AND FRESH ADULT BODY WEIGHT

The analysis of the fresh adult body weight and the wing size of the laboratory colony stable flies revealed a strong positive correlation (*r*^2^ = 0.8416; Figure S6), and this made it possible to predict the fresh adult body weight of the wild stable flies collected from various animal facilities. The predicted fresh adult body weight of the wild stable flies was 9.12 mg (± 0.11) and this was significantly greater (*p* < 0.0001) than the flies emerging from the horse manure in our assays, including the heaviest flies that started developing as larvae in 1 WHM (7.14 ± 0.02 mg).

### MICROBIAL COMMUNITY

The number of trimmed sequences and richness and diversity of the bacterial community of each sample of horse manure of different ages is depicted in **Table 2**. The number of good quality sequences was above 5000 for all samples with the exception of that of 1 WHM. The number of operational taxonomic units (OTUs) with a difference of 3% (genus level) showed that the bacterial community in the fresh manure was more diverse (1458 OTUs) than that of all older manures (all with less than 800 OTUs). The Shannon index (*H*′), Chao1, and ace indices also indicated greater bacterial diversity (*H*′) and richness (Chao1 and ace) of the fresh manure when compared to that of the aged manure (**Table 2**).

**Table 2:** Diversity and richness of the microbial community of horse manure at different age [fresh to 5 weeks old (5 WHM)].

Sample	No. of trimmed seqs	OTU 3%	*H*′ 3%	ace 3%	chao 3%
Fresh	6102	1458	6.24	2252.0	2145.51
1 WHM	3500	330	4.08	632.05	621.38
2 WHM	5470	625	5.18	861.17	854.29
3 WHM	5569	767	5.50	1100.39	1143.79
4 WHM	5127	685	4.63	959.56	880.85
5 WHM	6101	796	5.28	1131.01	1115.81

*OTU, operational taxonomic units observed with 3% cutoff (genus level) in distance units for describing and comparing communities. *H*′, nonparametric Shannon diversity index with 3% cutoff. ace, nonparametric richness abundance-coverage estimator with 3% cutoff. chao1, nonparametric richness estimator signifies the estimated minimum number of OTUs with a 3% cutoff*.

**FIGURE 5:**
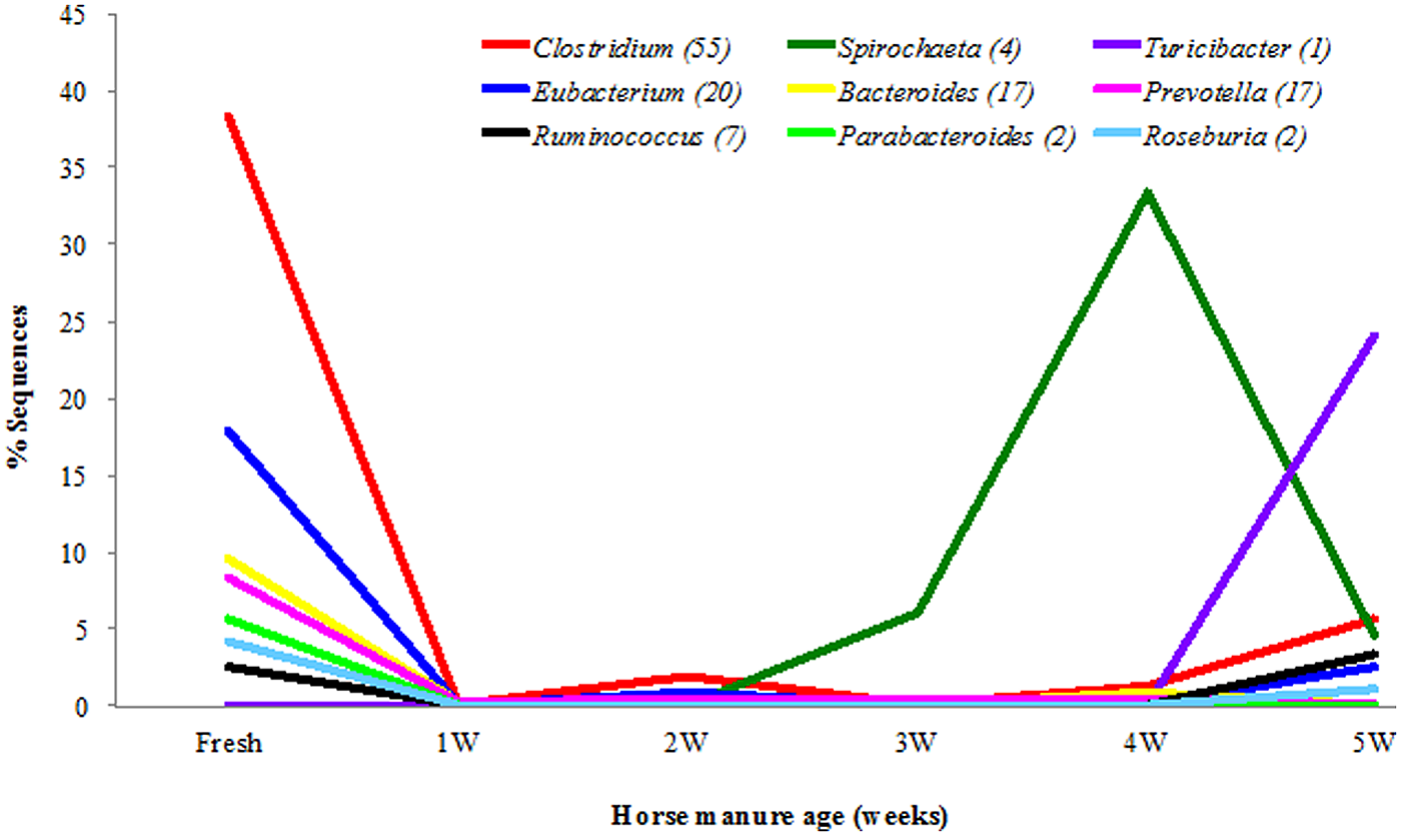
**Strictly anaerobic bacterial genera in different ages of the horse manure.** Genera (*n* = 9) with ≥1% of identified sequences on at least manure type.

**FIGURE 6:**
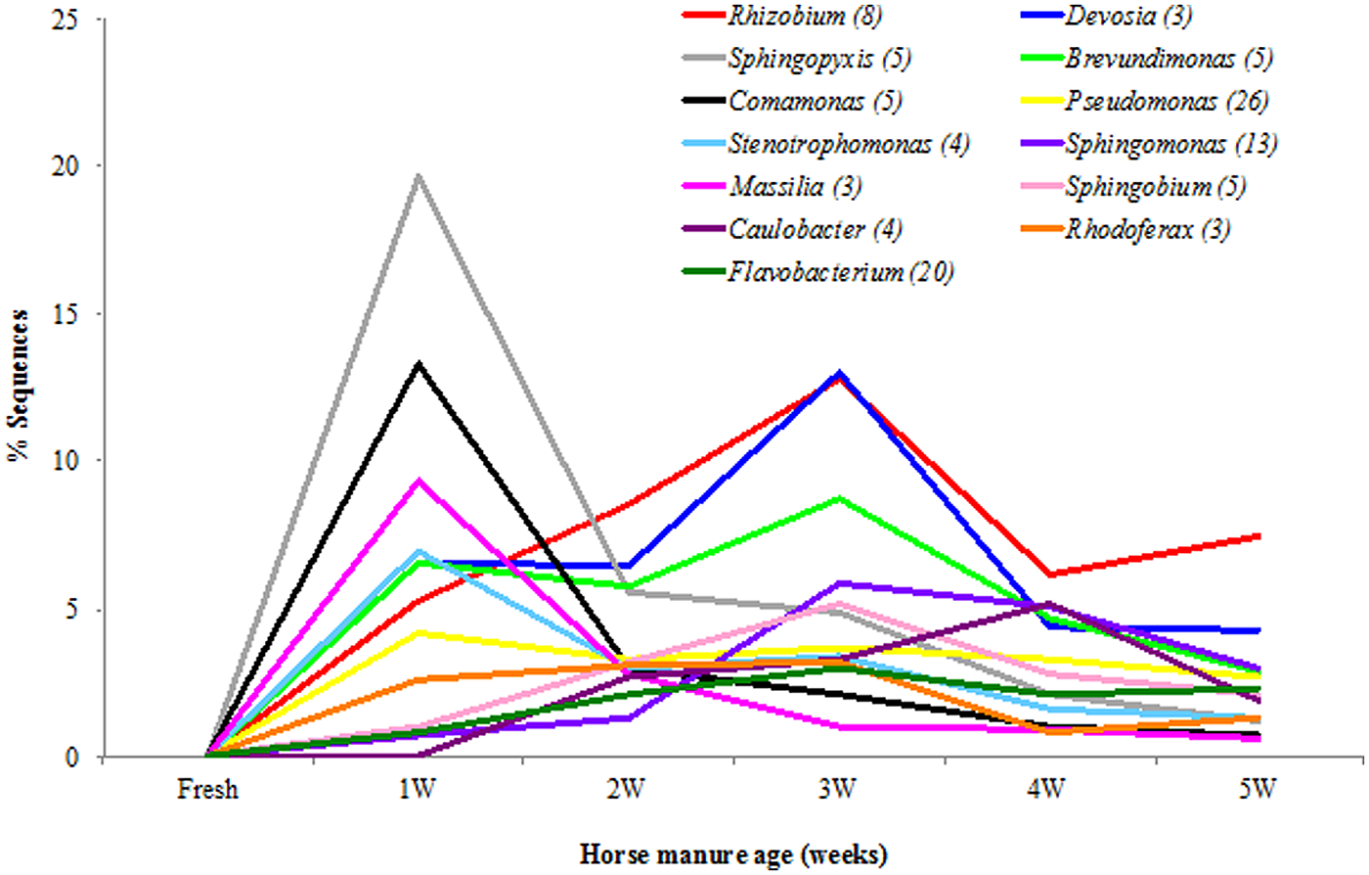
**Facultatively anaerobic and strictly aerobic bacterial genera in the horse manure of different age [fresh to 5 weeks old (5W)].** Genera (*n* = 13) with ≥1% of identified sequences on at least manure type.

Overall, in all samples of the horse manure, a total of 421 bacterial genera and 917 species were detected. The bacterial diversity of the horse manure at the genus level is shown in **Figure 5** and Table S2 (strict anaerobes) and **Figure 6** and Table S3 (facultative anaerobes and aerobes). Only bacterial taxa that represented ≥1% of identified sequences in at least one horse manure sample are reported. The fresh manure was clearly dominated by strictly anaerobic bacterial genera including, *Clostridium* (38.5%), *Eubacterium* (17.9%), *Bacteroides* (9.7%), *Prevotella* (8.3%), *Parabacteroides* (5.6%), and *Roseburia* (4.2%). Spirochete*s* were more common when the horse manure was 4 weeks old (33.4%) and *Turicibacter* when the manure was 5 weeks old (24.1%; **Figure 5**; Table S2) comparing to that of the fresh manure. A shift from strictly anaerobic bacteria in fresh manure to strict aerobes or facultative anaerobes in 1 WHM and older was evident (**Figures 5** and **6**).

Only few facultative anaerobic and aerobic bacterial taxa were detected when the manure was fresh (**Figure 6**; Table S3). Overall, *Rhizobium* (40.2%), *Devosia* (34.69%), *Sphingopyxis* (33.3%), *Brevundimonas* (28.5%), and *Comamonas* (20.1%) were the five most common genera in aging manure (1–5 WHM; Table S3). 2 and 3 WHM (that stimulated stable fly oviposition the most) was dominated by genera *Rhizobium* (21.4%), *Devosia* (19.5%), *Brevundimonas* (14.5%), *Sphingopyxis* (10.4%), and *Sphingobium* (8.4%; **Figure 6**; Table S3).

Overall, at the species level (1% difference among sequences), *Spirochaeta stenostrepta* (25.9%), *Turicibacter sanguinis* (18.1%), and *Clostridium symbiosum* (10.6%) were most common strict anaerobes (Table S4), and *Sphingopyxis witflariensis* (26.5%), *Devosia limi* (18.1%), and *Comamonas aquatica* (16.3%) were most common among the facultatively anaerobic and strictly aerobic bacteria (Table S5). The genus *Clostridium* was the most diverse and represented 55 species with *Clostridium symbiosum* and *Clostridium xylanolyticum* detected most frequently. *Devosia limi* (9.9%), *Rhizobium giardinii* (8.2%), *Brevundimonas diminuta* (7.5%), and *Sphingopyxis witflariensis* (7.3%) were the most commonly detected bacterial species in 2 and 3 WHM (Table S5).

## DISCUSSION

Many insects select the habitat for oviposition using semiochemical cues likely produced by the resident microbial community. The role of semiochemicals and/or microbial community in oviposition has been shown for mosquitoes (Ponnusamy et al., 2008a,b), sand flies (Peterkova-Koci et al., 2012), house flies (Lam et al., 2007), stable flies (Romero et al., 2006; Jeanbourquin and Guerin, 2007), and screwworm flies (Chaudhury et al., 2002, Chaudhury et al., 2012; reviewed by Leroy et al., 2011; Davis et al., 2013). The ability to select the appropriate oviposition site is critical for the survival and fitness of the next generation. Overall, the best substrates for larval development are considered those where larvae develop fast and adults are big (Gilles et al., 2008). Previously, we have shown that bacterial isolates stimulating stable fly oviposition also supported larval development and in reverse, those bacterial taxa that did not stimulate oviposition were inadequate for larval development (Romero et al., 2006).

Our results clearly show that fresh horse manure does not stimulate stable fly oviposition. It is likely that the bacterial community of fresh manure dominated by *Clostridia* and *Eubacteria* release cues that stable flies use to avoid oviposition and consequently competition with larvae of other insects such as horn flies and face flies. As the age of horse manure increased, the oviposition (% ovipositing flies and number of eggs deposited) increased; however, after 3 weeks of the aging process this trend reversed and significantly more eggs were laid again on control (2 WHM) manure. Metabolic products from bacteria such as spirochetes and *Turicibacter* in manure older than 3 weeks may indicate that the nutrients are depleted and do not support the larval development. This data corroborate Broce and Haas (1999) study where gravid females did not visit cattle manure piles while the manure was fresh, the visitations increased as the manure aged, and the first stable fly larvae were found in 2 week old cattle manure.

Interestingly, our data show that stable fly larvae can develop in fresh manure (60% survival) although adult females avoid oviposition in this substrate. It is important to point out that we collected very fresh horse feces before they got colonized by any arthropods and our experimental design did not allow any natural competition between stable fly larvae and other arthropods. When stable fly eggs were placed on 4 and 5 WHM, the survival of larvae to adult stage was very low (<20%), development time was significantly extended, and the adult body weight was significantly lower than that of flies from all other treatments. Although we did not analyze the nutritional composition of aging manure, it is very likely, that due to the microbial activity, nutrients in 4 and 5 WHM were depleted. Placement of eggs on 1–3 week old manure resulted in the greatest survival to adult stage although development time was extended and adult body weight decreased as the manure aged from 1 to 3 weeks old.

In our bioassays, all stable flies that emerged reached their critical body weight to start the metamorphosis but the quality or quantity of these nutrients was likely greatly reduced when the manure was older. This also explains why flies that emerged earlier from the same manure type were heavier than those that emerged later. Interestingly, analyses of the body weight of wild stable flies collected from dairy, feedlot, and horse facilities revealed that wild stable flies were heavier than those developing in the horse manure in our bioassays. The mean weight of wild stable flies was 9.12 mg comparing to 7.14 mg of the heaviest adults from 1 WHM suggesting that wild flies developed in more nutritious substrate than horse manure. Stable flies have a broad variety of substrates available and it is likely that horse manure is not the most suitable substrate for their larval development. On the other hand, it was shown that when given a choice stable flies laid more eggs to fresh horse manure than fresh cattle manure (Jeanbourquin and Guerin, 2007). However, our study showed that fresh manure is not a preferred oviposition substrate for stable flies and consequently assays comparing aged horse and cattle manure need to be conducted.

The bacterial diversity of the fresh manure in our study (Shannon index *H*′ = 6.24) was comparable to that (6.7) observed in Shepherd et al. (2012) and higher than in other species; beef cattle feces (*H*′ = 4.9; Durso et al., 2010), pig feces (*H*′ = 3.2; Lamendella et al., 2011), and human feces (*H*′ = 4.0; Andersson et al., 2008). It has been suggested that the higher bacterial diversity in the horse feces is due to a high fiber diet (Shepherd et al., 2012). In our study, the bacterial community of fresh horse manure was composed mainly by *Firmicutes*, *Bacteroidetes*, *Proteobacteria*, and *Spirochaetes* and these typically dominate the fresh fecal bacterial community of other mammals (Ozutsumi et al., 2005; Durso et al., 2010; Lamendella et al., 2011).

After exposure to air, a shift from anaerobic bacteria to aerobic and facultative anaerobic bacteria was expected and observed. The manure 2 and 3 weeks old (most attractive for stable fly oviposition) was dominated by Alphaproteobacteria including *Rhizobium*, *Devosia, Brevundimonas*, *Sphingopyxis*, and *Sphingobium*. *Rhizobium* is a common soil bacterium capable of nitrogen fixation and found in association with plants (Brenner et al., 2005). The diet of horses used in this study included alfalfa hay and that is most likely the source of *Rhizobium* spp. detected in the horse feces. *Devosia* spp. are Gram-negative rods, also commonly found in soil (Brenner et al., 2005; Yoo et al., 2006). *Devosia* spp. are not able to hydrolyze gelatin or starch but they can degrade urea and poorly reduce nitrate to nitrite (Brenner et al., 2005). *Brevundimonas* spp. are aquatic (Brenner et al., 2005), non-fermenting gram-negative bacilli that have been reported to be rare opportunistic pathogens of immunocompromised hosts (Lee et al., 2011). All species can grow utilizing pyruvate, and most isolates can also use amino acids (glutamate and proline) and organic acids (acetate, butyrate, fumarate, and succinate). All *Brevundimonas* spp. can use glucose, galactose, maltose, and starch (Brenner et al., 2005). Metabolic products (volatile and non-volatile) of these bacteria need to be analyzed and tested for their oviposition stimulation and significance in larva development of stable flies. It is also possible that other less abundant bacterial taxa in 2 and 3 WHM are important in stable fly behavior and fitness. It is important to point out several limitations of the bacterial community analysis. (1) The analysis was done only on one set of samples of the aging manure and further studies are needed to provide the additional data from biological replicates; (2) Despite our efforts to homogenize each sample, 1 g of each sample used for DNA extraction may not be representative enough for the entire substrate; (3) The control samples (2 week old manure) for oviposition and larval development assays were generated by collecting fresh manure for each corresponding aged manure. Although the fresh manure was always collected from the same group of horses on the same diet, it is not identical control samples across all assays; (4) In the field, other factors such as natural arthropod populations will likely contribute to changes of aging animal manure.

The identification of the attractive natural substrate for oviposition is of great relevance for improving stable fly management. The preferred substrate (type and age) can help predict when adult flies are emerging and when control measures need to be applied. Weather conditions also likely affect stable fly development and temperatures well above 26∘C may result in faster decomposition of horse manure allowing stable flies to oviposit on fresher manure. Temperatures below 26∘C may have the opposite effect on manure decomposition and stable fly oviposition and larval development. Consequently, timing of management of the larval habitat could be adjusted based on variations in temperature. Currently, control of stable flies in animal facilities relies on sanitation and reducing the larva developmental sites (Broce, 2006). The schedule of this sanitation is not well established. A fortnightly cleaning schedule in Nebraska feedlots during the stable fly season showed 50% reduction in stable fly populations (Thomas et al., 1996). Typical guidelines recommend removing manure from dairies and feedlots on weekly basis and then piled it up and cover with a heavy-duty plastic sheets or spread on agricultural fields (Hogsette, 1986). This practice is laborious and time consuming and thus not commonly adopted. Based on our study, at temperatures around 26∘C, horse manure management based on disrupting larval development, should focus on 2–4 WHM and could be done twice a month to break the stable fly life cycle and to prevent a build-up of large populations of stable flies.

In conclusion, this is the first study analyzing the bacterial community in the horse manure during the aging process and its correlation to stable fly behavior and fitness. The preference of stable flies to oviposit on 2 and 3 WHM and the successful larval development in this manure suggest that the resident microbial community generates the cues for stable fly oviposition and it is also important for the larval development. The identification of the semiochemical cues and their specific microbial origin as well as the basis of the larval dependence on microorganisms remain to be studied. Future investigations of the stable fly – microbial associations should lead to development of novel approaches for management of this insect pest based on (1) modification of the bacterial community of animal manure, and/or (2) development of novel attractants and repellents, and/or (3) paratransgenic approach.

## Conflict of Interest Statement

The authors declare that the research was conducted in the absence of any commercial or financial relationships that could be construed as a potential conflict of interest.
