# Supplementary material for: Temporal changes in the bacterial community of animal feces and their correlation with stable fly oviposition, larval development, and adult fitness
Source: Front Microbiol. 2014 Nov 10;5:590. doi: 10.3389/fmicb.2014.00590 (PMC4226234; doi:10.3389/fmicb.2014.00590)
Supplement: Supplementary file 1 [file Data_Sheet_1.DOCX]

***Supplementary Material***

**Temporal changes in the microbial community of animal feces greatly affect stable fly oviposition, larval development, and adult fitness**

Thais A. Albuquerque^1^ and Ludek Zurek^1,2*^

^1^Department of Entomology

^2^Department of Diagnostic Medicine and Pathobiology

Kansas State University

Manhattan, KS, USA

*Correspondence:

Ludek Zurek

^1^Department of Entomology

College of Agriculture

^2^Department of Diagnostic Medicine and Pathobiology

College of Veterinary Medicine

123 Waters Hall

Kansas State University

Manhattan, KS 66506, USA

Phone: 785 532 4731

Fax: 785 532 6232

Email: [lzurek@ksu.edu](mailto:lzurek@ksu.edu)

Table S1. Oviposition (% of flies) of individual stable flies on aging (fresh to 5 weeks old [5WHM]) and control (2WHM) horse

manure

|  | | **% Flies (95% CI)*** | | | | | |
| --- | --- | --- | --- | --- | --- | --- | --- |
|  | **Fresh** | | **1WHM** | **2WHM** | **3WHM** | **4WHM** | **5WHM** |
| Aging | 0 (-2-12)^b^ | | 12.9 (5-27)^a^ | 23.4 (13-40)^a^ | 20.8 (8-39)^a^ | 2.8 (-1-16) ^a^ | 3.9 (-1-17)^a^ |
| Control | 100 (88-102)^a^ | | 38.2 (25-54)^ab^ | 10.9 (4-25)^a^ | 48.2 (32-68)^a^ | 57.8 (41-72)^b^ | 75.8 (62-90)^b^ |
| Both plates | 0 (-2-12)^b^ | | 49 (34-64)^b^ | 65.8 (49-78)^b^ | 31 (17-50)^a^ | 39.4 (26-56)^b^ | 20.4 (8-35)^a^ |

Different letters indicate significant differences (p<0.05) among the three different choices within the same experiment. *CI = confidence interval.

**Table S2**. Genera of anaerobic bacteria (%) detected in different ages of the horse manure.

| **genus (no. of species)** | **Fresh** | **1W** | **2W** | **3W** | **4W** | **5W** | **Total** |
| --- | --- | --- | --- | --- | --- | --- | --- |
| ***Clostridium* (55)** | 38.47 | 0.13 | 1.93 | 0.15 | 1.36 | 5.62 | **47.66** |
| ***Spirochaeta* (4)** | 0.01 | 0.00 | 0.00 | 6.18 | 33.42 | 4.68 | **44.30** |
| ***Turicibacter* (1)** | 0.00 | 0.00 | 0.18 | 0.00 | 0.03 | 24.13 | **24.33** |
| ***Eubacterium* (20)** | 17.93 | 0.13 | 0.89 | 0.03 | 0.17 | 2.65 | **21.80** |
| ***Bacteroides* (17)** | 9.70 | 0.26 | 0.35 | 0.11 | 1.02 | 0.08 | **11.52** |
| ***Prevotella* (17)** | 8.27 | 0.36 | 0.44 | 0.51 | 0.44 | 0.15 | **10.17** |
| ***Ruminococcus* (7)** | 2.56 | 0.00 | 0.11 | 0.00 | 0.00 | 3.46 | **6.13** |
| ***Parabacteroides* (2)** | 5.64 | 0.00 | 0.03 | 0.03 | 0.03 | 0.00 | **5.72** |
| ***Roseburia* (2)** | 4.16 | 0.10 | 0.05 | 0.00 | 0.00 | 1.12 | **5.43** |
| ***Opitutus* (1)** | 0.00 | 0.00 | 0.03 | 0.06 | 1.24 | 1.86 | **3.18** |
| ***Sporobacter* (1)** | 2.37 | 0.00 | 0.03 | 0.05 | 0.00 | 0.21 | **2.66** |
| ***Treponema* (8)** | 1.80 | 0.12 | 0.03 | 0.17 | 0.39 | 0.10 | **2.60** |
| ***Porphyromonas* (5)** | 0.03 | 0.00 | 2.55 | 0.00 | 0.00 | 0.00 | **2.58** |
| ***Pseudobutyrivibrio* (1)** | 2.42 | 0.00 | 0.14 | 0.00 | 0.00 | 0.00 | **2.56** |
| ***Fusobacterium* (4)** | 0.01 | 0.43 | 1.54 | 0.00 | 0.00 | 0.43 | **2.41** |
| ***Anaerovorax* (1)** | 1.84 | 0.00 | 0.16 | 0.00 | 0.00 | 0.00 | **2.01** |
| ***Allobaculum* (1)** | 0.00 | 0.00 | 1.72 | 0.00 | 0.00 | 0.14 | **1.86** |

Numbers are percentage of identified sequences.

Table S3. Genera of facultative and aerobic bacteria (%) detected in the horse manure of different age (fresh to 5 weeks [5W] old).

| **genus (no. of species)** | **Fresh** | **1W** | **2W** | **3W** | **4W** | **5W** | **Total** |
| --- | --- | --- | --- | --- | --- | --- | --- |
| ***Rhizobium* (8)** | 0.00 | 5.24 | 8.56 | 12.81 | 6.17 | 7.41 | **40.20** |
| ***Devosia* (3)** | 0.00 | 6.53 | 6.46 | 13.01 | 4.42 | 4.28 | **34.69** |
| ***Sphingopyxis* (5)** | 0.00 | 19.62 | 5.53 | 4.89 | 2.05 | 1.16 | **33.26** |
| ***Brevundimonas* (5)** | 0.00 | 6.56 | 5.76 | 8.71 | 4.62 | 2.88 | **28.54** |
| ***Comamonas* (5)** | 0.00 | 13.33 | 2.98 | 2.08 | 1.05 | 0.65 | **20.09** |
| ***Pseudomonas* (26)** | 0.03 | 4.16 | 3.24 | 3.69 | 3.23 | 2.65 | **17.00** |
| ***Stenotrophomonas* (4)** | 0.00 | 6.99 | 3.02 | 3.35 | 1.55 | 1.29 | **16.20** |
| ***Sphingomonas* (13)** | 0.00 | 0.65 | 1.29 | 5.89 | 5.06 | 2.96 | **15.86** |
| ***Massilia* (3)** | 0.00 | 9.36 | 2.76 | 1.03 | 0.90 | 0.60 | **14.65** |
| ***Sphingobium* (5)** | 0.00 | 1.00 | 3.21 | 5.20 | 2.83 | 2.12 | **14.36** |
| ***Caulobacter* (4)** | 0.00 | 0.00 | 2.70 | 3.30 | 5.20 | 1.88 | **13.08** |
| ***Rhodoferax* (3)** | 0.00 | 2.57 | 3.12 | 3.16 | 0.75 | 1.25 | **10.84** |
| ***Flavobacterium* (20)** | 0.00 | 0.81 | 2.04 | 2.99 | 2.05 | 2.33 | **10.22** |
| ***Haemophilus* (4)** | 0.18 | 0.33 | 9.18 | 0.00 | 0.00 | 0.00 | **9.69** |
| ***Rhodobacter* (2)** | 0.00 | 5.92 | 0.93 | 1.03 | 0.53 | 0.26 | **8.68** |
| ***Phenylobacterium* (4)** | 0.00 | 0.20 | 1.27 | 2.16 | 2.32 | 1.55 | **7.50** |
| ***Phyllobacterium* (3)** | 0.00 | 0.00 | 0.09 | 2.50 | 2.43 | 2.22 | **7.24** |
| ***Rubrivivax* (1)** | 0.00 | 0.00 | 0.14 | 0.34 | 1.99 | 4.64 | **7.11** |
| ***Pedobacter* (8)** | 0.09 | 1.60 | 0.93 | 1.93 | 0.93 | 1.05 | **6.54** |
| ***Dyadobacter* (3)** | 0.00 | 0.00 | 1.03 | 2.54 | 1.27 | 1.03 | **5.86** |
| ***Wautersiella* (1)** | 0.00 | 0.48 | 0.09 | 2.79 | 1.55 | 0.93 | **5.83** |
| ***Terrimonas* (1)** | 0.00 | 0.00 | 0.11 | 1.10 | 1.89 | 2.33 | **5.42** |
| ***Paracoccus* (9)** | 0.00 | 3.28 | 0.66 | 0.78 | 0.41 | 0.15 | **5.29** |

| ***Janthinobacterium* (2)** | 0.00 | 4.08 | 0.48 | 0.18 | 0.00 | 0.32 | **5.06** |
| --- | --- | --- | --- | --- | --- | --- | --- |
| ***Algoriphagus* (8)** | 0.26 | 0.16 | 0.70 | 1.28 | 1.34 | 1.18 | **4.92** |
| ***Alysiella* (1)** | 0.00 | 0.00 | 4.78 | 0.00 | 0.00 | 0.00 | **4.78** |
| ***Streptococcus* (14)** | 1.02 | 0.54 | 2.51 | 0.00 | 0.58 | 0.04 | **4.69** |
| ***Novosphingobium* (6)** | 0.00 | 0.57 | 0.93 | 1.32 | 1.14 | 0.72 | **4.67** |
| ***Lactobacillus* (8)** | 0.13 | 0.03 | 0.90 | 0.05 | 1.65 | 1.81 | **4.58** |
| ***Acinetobacter* (6)** | 0.00 | 2.86 | 1.18 | 0.32 | 0.05 | 0.15 | **4.56** |
| ***Moraxella* (1)** | 3.06 | 0.00 | 0.65 | 0.03 | 0.14 | 0.17 | **4.05** |
| ***Mesorhizobium* (5)** | 0.00 | 0.00 | 0.25 | 1.53 | 0.97 | 1.25 | **4.00** |
| ***Ochrobactrum* (5)** | 0.00 | 0.13 | 0.36 | 1.67 | 0.74 | 0.68 | **3.59** |
| ***Xanthomonas* (5)** | 0.00 | 1.32 | 0.69 | 0.55 | 0.41 | 0.21 | **3.18** |
| ***Chloroflexus* (1)** | 0.00 | 0.00 | 0.05 | 0.23 | 1.14 | 1.61 | **3.02** |
| ***Actinobacillus* (4)** | 0.00 | 0.00 | 2.90 | 0.00 | 0.00 | 0.00 | **2.90** |
| ***Neisseria* (5)** | 0.00 | 0.00 | 2.68 | 0.00 | 0.03 | 0.00 | **2.71** |
| ***Chromohalobacter* (1)** | 0.00 | 0.00 | 0.03 | 0.26 | 0.53 | 1.62 | **2.43** |
| ***Gemella* (1)** | 0.00 | 0.00 | 2.22 | 0.00 | 0.00 | 0.00 | **2.22** |
| ***Conchiformibius* (1)** | 0.00 | 0.12 | 1.88 | 0.00 | 0.00 | 0.00 | **2.00** |
| ***Bergeyella* (1)** | 0.00 | 0.00 | 1.57 | 0.00 | 0.00 | 0.00 | **1.57** |

Numbers are percentage of identified sequences.

Table S4. Species diversity of strictly anaerobic bacteria (%) representing ≥ 1% of identified sequences in ≥ 1 type of horse

manure.

| **name** | **Fresh** | **1WHM** | **2WHM** | **3WHM** | **4WHM** | **5WHM** | **Total** |
| --- | --- | --- | --- | --- | --- | --- | --- |
| ***Bacteroidales oral*** | 5.18 | 0.17 | 0.07 | 0.07 | 0.09 | 0.03 | **5.61** |
| ***Bacteroides capillosus*** | 1.61 | 0.06 | 0.04 | 0.00 | 0.04 | 0.00 | **1.76** |
| ***Clostridium algidixylanolyticum*** | 1.19 | 0.00 | 0.00 | 0.00 | 0.00 | 0.00 | **1.19** |
| ***Clostridium bolteae*** | 2.21 | 0.00 | 0.02 | 0.00 | 0.00 | 0.05 | **2.28** |
| ***Clostridium celerecrescens*** | 1.43 | 0.00 | 0.00 | 0.02 | 0.00 | 1.07 | **2.52** |
| ***Clostridium hathewayi*** | 2.35 | 0.00 | 0.00 | 0.00 | 0.02 | 0.03 | **2.41** |
| ***Clostridium jejuense*** | 1.04 | 0.00 | 0.02 | 0.00 | 0.02 | 0.00 | **1.08** |
| ***Clostridium methylpentosum*** | 1.92 | 0.00 | 0.02 | 0.04 | 0.00 | 0.00 | **1.98** |
| ***Clostridium nexile*** | 1.08 | 0.00 | 0.00 | 0.00 | 0.00 | 0.00 | **1.08** |
| ***Clostridium orbiscindens*** | 1.25 | 0.00 | 0.02 | 0.00 | 0.00 | 0.01 | **1.28** |
| ***Clostridium saccharolyticum*** | 1.01 | 0.00 | 0.00 | 0.00 | 0.00 | 0.31 | **1.32** |
| ***Clostridium symbiosum*** | 10.13 | 0.03 | 0.21 | 0.02 | 0.04 | 0.12 | **10.55** |
| ***Clostridium xylanolyticum*** | 5.33 | 0.00 | 0.00 | 0.00 | 0.00 | 0.00 | **5.33** |
| ***Eubacterium rectale*** | 2.16 | 0.00 | 0.00 | 0.00 | 0.00 | 0.00 | **2.16** |
| ***Eubacterium ruminantium*** | 9.12 | 0.09 | 0.07 | 0.02 | 0.11 | 0.24 | **9.66** |
| ***Eubacterium siraeum*** | 2.73 | 0.03 | 0.00 | 0.00 | 0.02 | 0.00 | **2.78** |
| ***Parabacteroides goldsteinii*** | 4.60 | 0.00 | 0.02 | 0.02 | 0.02 | 0.00 | **4.66** |
| ***Prevotella bivia*** | 3.09 | 0.03 | 0.00 | 0.02 | 0.00 | 0.00 | **3.14** |
| ***Prevotella bryantii*** | 2.04 | 0.11 | 0.07 | 0.14 | 0.09 | 0.05 | **2.51** |
| ***Roseburia intestinalis*** | 3.52 | 0.09 | 0.04 | 0.00 | 0.00 | 0.80 | **4.45** |
| ***Ruminococcus obeum*** | 0.15 | 0.00 | 0.09 | 0.00 | 0.00 | 2.23 | **2.47** |
| ***Spirochaeta americana*** | 0.00 | 0.00 | 0.00 | 0.98 | 6.14 | 0.76 | **7.88** |
| ***Spirochaeta stenostrepta*** | 0.00 | 0.00 | 0.00 | 3.83 | 19.49 | 2.61 | **25.94** |
| ***Turicibacter sanguinis*** | 0.00 | 0.00 | 0.14 | 0.00 | 0.02 | 17.88 | **18.05** |

Numbers are percentage of identified sequences.

Table S5. Species diversity of facultatively anaerobic bacteria and strictly aerobic bacteria (%) representing ≥ 1% of identified sequences in ≥ 1 type of horse manure.

| **name** | **Fresh** | **1WHM** | **2WHM** | **3WHM** | **4WHM** | **5WHM** | **Total** |
| --- | --- | --- | --- | --- | --- | --- | --- |
| ***Brevundimonas diminuta*** | 0.00 | 5.90 | 3.61 | 3.86 | 1.23 | 0.77 | **15.38** |
| ***Brevundimonas nasdae*** | 0.00 | 0.03 | 0.96 | 1.81 | 1.43 | 0.73 | **4.96** |
| ***Caulobacter vibrioides*** | 0.00 | 0.00 | 2.02 | 2.26 | 3.65 | 1.25 | **9.18** |
| ***Comamonas aquatica*** | 0.00 | 11.23 | 2.24 | 1.60 | 0.75 | 0.47 | **16.29** |
| ***Devosia limi*** | 0.00 | 4.62 | 3.56 | 6.37 | 1.93 | 1.62 | **18.11** |
| ***Devosia riboflavina*** | 0.00 | 1.22 | 1.61 | 3.06 | 1.01 | 0.63 | **7.52** |
| ***Flavobacterium mizutaii*** | 0.00 | 0.06 | 0.24 | 1.12 | 0.22 | 0.23 | **1.85** |
| ***Massilia timonae*** | 0.00 | 8.43 | 2.24 | 0.82 | 0.68 | 0.43 | **12.60** |
| ***Pseudomonas lini*** | 0.00 | 2.04 | 0.92 | 0.53 | 0.17 | 0.07 | **3.74** |
| ***Rhizobium etli*** | 0.00 | 0.20 | 0.43 | 1.77 | 1.37 | 1.36 | **5.12** |
| ***Rhizobium giardinii*** | 0.00 | 3.72 | 4.32 | 3.91 | 1.08 | 1.65 | **14.68** |
| ***Rhizobium huautlense*** | 0.00 | 0.14 | 1.80 | 4.05 | 1.98 | 2.04 | **10.01** |
| ***Rhodoferax ferrireducens*** | 0.00 | 2.32 | 2.55 | 2.47 | 0.60 | 0.92 | **8.86** |
| ***Sphingobium herbicidovorans*** | 0.00 | 0.90 | 2.18 | 3.40 | 2.07 | 1.39 | **9.94** |
| ***Sphingomonas koreensis*** | 0.00 | 0.03 | 0.18 | 1.90 | 2.35 | 1.53 | **5.99** |
| ***Sphingopyxis witflariensis*** | 0.00 | 17.68 | 4.39 | 2.90 | 0.90 | 0.62 | **26.49** |
| ***Stenotrophomonas rhizophila*** | 0.00 | 4.86 | 1.91 | 2.17 | 0.79 | 0.68 | **10.41** |

Numbers are percentage of identified sequences.

Figure S1. Oviposition (number of eggs) of a group of stable flies (n=30 per assay) on aging and 2 week old horse manure (2WHM) (control).

Aging manure (white bar) is fresh at week 0 and ages progressively to 5 weeks old. Stars indicate a significant difference (p<0.05) between the two choices within the same week. Error bars are standard error of mean.

Figure S2. Stable fly daily emergence from the horse manure of different ages and artificial medium (control).

n=1500.

Figure S3. The daily mean weight of stable flies freshly emerged from horse manure of different ages and from the artificial medium (control). (n=919, 1262, 1190, 1097, 224, 32, and 2987, respectively).

Figure S4. Correlation between stable fly development time and the substrate age (fresh to 5 weeks old [5W]) at oviposition.

Bars are standard error of mean. (r^2^= 0.82; n= 919, 1262, 1190, 1097, 224, and 32, respectively).

Figure S5. Correlation between the stable fly fresh adult body weight (mg) and the horse manure age (fresh to 5 weeks old [5W]) at oviposition.

Bars are standard error of mean. r^2^= -0.72. (n= 919, 1262, 1190, 1097, 224, and 32, respectively).

**Figure S6.** Correlation of the stable fly wing size (mm) and the fresh adult body weight (mg).

n= 140. r^2^= 0.84.
